# Investigating the Effect of Algal Inclusions in Broiler Chickens

**DOI:** 10.3390/life15040670

**Published:** 2025-04-17

**Authors:** Hanan Al-Khalaifah, Afaf Al-Nasser, Tahani Al-Surrayai

**Affiliations:** Environment and Life Sciences Research Center, Kuwait Institute for Scientific Research, P.O. Box 24885, Safat 13109, Kuwait

**Keywords:** algae, antioxidant status, broiler, lipid, poultry

## Abstract

There is growing interest in adding marine algae to poultry feed rations. The aim of the present study is to examine the effect of various inclusions of *Sargassum* sp., *Gracilaria* sp., and *Spirulina* sp. on productive performance, serum proteins, liver proteins, meat quality, and antioxidant activity in broiler chickens. There were seven dietary treatments (TRTs) as follows: TRT 1 was the control group without algae; TRT 2 was enriched with *Sargassum* sp. at 1% of the diet; TRT 3 with *Sargassum* sp. at 2% of the diet; TRT 4 with *Gracilaria* sp. at 0.5% of the diet; TRT 5 with *Gracilaria* sp. at 1% of the diet; TRT 6 with *Spirulina* sp. at 5% of the diet; TRT 7 with *Spirulina* sp. at 7.5% of the diet. Each treatment was replicated ten times, with 17 birds per replicate, and the analyses were performed in triplicates. Results of the feed rations proximate analyses revealed that the formulated diets contained the required amounts of protein, fat, fiber, ash, and moisture, to be fed to the broiler chickens. There was no effect of marine algae on the production performance parameters of the birds. All the enriched birds performed normally as the control group. It was shown that enriching the broiler diet with 1% *Sargassum* sp. Induced an increase in the total serum proteins, while *Gracilaria* sp. algal inclusion reduced the total serum proteins, compared to the control group. At five weeks of age, enriching broiler diets with 5% *Spirulina* sp. resulted in a higher concentration of total serum protein-C than the control group and the group enriched with 7.5% inclusion. Enriching the diet of 3- wks old broilers with *Sargassum* sp. at 2% elevated the proportions of serum LFABP. The maximum proportion of omega 6 fatty acid (∑n-6) was observed in the group of birds that received the control diet, while the proportion of omega 3 fatty acid (∑n-3) was highest in the algae-enriched groups. The results showed that all algal inclusions lowered the ratio of omega-6 to omega-3 fatty acids (∑n-6:∑n-3). Enriching broiler chickens with *Spirulina* at 5% and 7.5% increased the HDL concentration, compared to the control group. The palatability of meat for color, texture, flavor, appearance, smell, and overall acceptability was not negatively affected by algal inclusions. All algal inclusions enhanced the anti-oxidative status of broilers and lipid oxidative stability of the stored feed rations. In general, it can be concluded that marine algal inclusions showed no effect on the productive performance of the broiler chickens and can be used without any detrimental effects in poultry feed rations.

## 1. Introduction

A significant global challenge facing the poultry industry is the cost and quality of feed, which makes up about 60–70% of the total operational costs. Recently, there has been growing interest in utilizing natural resources to partially replace the corn and soybean in poultry feed for protein and energy. Additionally, there is a focus on incorporating effective ingredients to enhance poultry health and productivity. Examples of these effective ingredients include algae, probiotics, and native plants [1,2,3,4].

Macroalgae, commonly referred to as seaweed, are multicellular organisms predominantly found in saltwater or freshwater. They are classified into three types based on their pigmentation: brown seaweed (Phaeophyceae), red seaweed (Rhodophyceae), and green seaweed (Chlorophyceae). They are very rich in beneficial metabolites (pigments, carotenoids, phlorotannins, polyunsaturated fatty acids, agar, alginate and carrageenan) and minerals (iodine, zinc, sodium, calcium, manganese, iron, and selenium), being considered as a natural source of additives that can substitute for antibiotics in various animals. The nutritional value of microalgae can vary significantly between species due to differences in cell size and structure, bioactive components, and growing conditions. Despite these variations, the primary nutritional components in algae generally include protein (6–63%), carbohydrates (8–64%), and lipids (2–50%) [5,6]. Algae possess antioxidant, immune-enhancing, antimicrobial, and anti-inflammatory properties. The antioxidative nature of a large number of algae has been found to be beneficial in the prevention and cure of cancer. Some of the powerful antioxidants found are polyphenols, phycobiliproteins, and vitamins. It is proven that these antioxidants can prevent cancer by regressing the oxidative processes that lead to carcinogenesis. They also help in fighting diseases such as chronic inflammation, cardiovascular diseases, atherosclerosis, and ageing processes [7,8,9].

Incorporating marine algae into poultry feed rations can serve as a partial substitute for traditional protein sources, enhancing both production performance and the overall health of poultry flocks. Algae could be added to poultry feed in proportions of up to 2 to 5% without causing any adverse effect on the performance or taste of poultry meat. However, some authors have reported the beneficiary effect of using marine algae at 10% of chickens’ diet [10,11,12]. Recent research studies have confirmed that algae can be used as effectively as fish oil to enrich poultry meat with n-3 polyunsaturated fatty acids (PUFAs) [1,13].

Marine algae have been described as affecting the proportions of physiological activities such as proportions of serum proteins. These proteins are primarily synthesized in the liver and play a crucial role in various bodily functions. They help maintain blood volume by exerting colloidal osmotic pressure, regulate blood pH through buffering, and transport hormones and drugs throughout the bloodstream. Additionally, they are involved in blood coagulation to prevent excessive bleeding, act as enzymes to catalyze chemical reactions, and regulate metabolism through hormonal actions. They also contribute to the body’s defense mechanisms against foreign agents. Protein-C and protein-S are blood proteins that regulate clot formation, preventing excessive clotting. Deficiency or dysfunction of these proteins can be inherited or acquired due to conditions like liver disease, kidney disease, severe infections, or cancer [14]. It is reported by some studies in the literature that enriching poultry feed rations with algal inclusions may influence the concentration of serum proteins that could affect the productive performance in poultry. For example, Abd El-Hady et al. [15] studied the effect of *Spirulina* sp. on productive performance and serum proteins in broiler chickens. The authors elected to feed the broilers on diets containing 0, 3, and 6% of *Spirulina* sp. and reported that serum total lipid, cholesterol, and triglyceride concentrations in the diet containing 3 or 6% *Spirulina* sp. decreased in comparison with the control group. In the same study, the concentration of high-density lipoprotein was increased, while the low-density lipoprotein concentration decreased with the algal supplementation. Serum protein concentrations had increased in the group of birds that received 3 or 6% *Spirulina* sp. compared to the control group.

*Sargassum* sp. are naturally and locally occurring brown algae, which have been used extensively in various industries and for nutritional purposes [16]. They generally inhabit shallow waters and coral reefs. Floating populations of these algae are also observed in some cases. *Sargassum* sp. are thought to impact the performance and health of animals, including enhancing the productive performance of chickens. These marine algae may offer nutritional benefits that support growth, the immune function, and overall well-being in poultry [17,18,19]. These species are also claimed to improve the fat content in broilers by reducing cholesterol levels in the produced meat, eggs, and their blood serum [20,21,22,23]. In addition, the nutritious value of algae species and their utilization as feed have been registered in many studies [24]. Brown algae are rich in minerals, vitamins, essential amino acids, polysaccharides, ome-ga-3 and omega-6 fatty acids, and sterols [23,25]. Erum et al. [26] reported that broiler chickens fed a diet enriched with increasing levels of air-dried and ground *Sargassum muticum* showed reduced fat pad development. The carcass quality was enhanced due to the reduction in fat in the enriched group. In the control group with no algae added, the color of fat pads and meat was yellowish, compared to the group enriched with 10% of the algae. With 15% of *S. muticum*, there were no fat pads and the meat color was reddish. In addition, Kumar [27] investigated the effect of *Sargassum* sp. at 1%, 2%, 3% and 4% of diet on meat parameters including color, flavor, tenderness, juiciness, taste, and weight. Meat from broilers fed with basal diet with the addition of 1% or 2% of *Sargassum* sp. received the highest scores. The inclusion of this brown seaweed at all levels led to enhancements in carcass traits, such as the weight of the legs, breast, thighs, and overall dressing percentage. The most significant supplementary effects were observed at *Sargassum* doses of 1% and 2%, attributed to the seaweed’s rich chemical composition, including minerals, vitamins, polyunsaturated fatty acids, essential amino acids, sterols, and polysaccharides like fucoidan [27]. In another study of El-Deek et al. [28], the authors reported that enriching the feed rations of broiler chickens with 2%, 4% and 6% of thermally treated *Sargassum dentifebium* enhanced the dressing percentage, compared to the control group.

The red algae of the genus *Gracilaria* (*Rhodophyta*) are considered excellent candidates for intensive culture in aquaculture due to their ability to achieve high yields and produce economically valuable products. These algaes how a greater level of biodiversity and many of these algae are rich in protein and are used in dried form as protein sources in formulated animal feed [29]. Recently, Jiang et al. [30] investigated the effects of dietary supplementation with *Gracilaria* sp. polysaccharides on the growth performance, antioxidant capacity, immune function, and meat quality of broiler chickens. The author used 0 (control), 1000, 2000, or 4000 mg/kg diet of polysaccharides. They reported that dietary supplementation with 2000 mg/kg of algal polysaccharides increased the average daily weight gain from days 0 to42 and decreased the feed efficiency. Broilers fed supplemented diets had increased serum superoxide dismutase, glutathione peroxidase, serum catalase activity, and catalase activities in the liver, whereas liver malondialdehyde concentration was decreased. In the same study, the authors also reported broilers had increased serum immunoglobulin (Ig) A, IgG, interleukin (IL)-6, IL-1β, IL-10, and interferon-γ concentrations in the supplemented group compared to the control group [30].

*Spirulina* is a green microalga widely used in animal nutrition for its high content of protein, minerals, vitamins, amino acids, and essential fatty acids [15]. It also contains thiamine, riboflavin, pyridoxine, vitamin B12, vitamin C, carotenoids, calcium, iron, and antioxidants [31], which makes it a good source of effective nutrients for animal production. *Spirulina* was used in poultry production to enrich the immune and health status of the flocks via an increasing T-cell activity defense mechanism [32]. In addition, Sugiharto [33] reported that the microalga *Spirulina* enhances the growth performance of broiler chickens, improves immune responses, intestinal morphology, and the microbial ecosystem, and boosts the antioxidative status of broilers.

Dried full-fat *Spirulina* algae, with an energy value equivalent to 90% of corn (2839 kcal TMEn/kg), offers a rich source of crude protein, comprising 76% of its content, along with essential amino acids. The research has indicated that up to 16% of dried *Spirulina* algae can be successfully incorporated into a broiler starter diet without adversely affecting the production performance of chicks [34].

The purpose of the current study is to investigate the effect of *Sargassum* sp., *Gracilaria* sp., and *Spirulina* sp. inclusions on the productive performance of broiler chickens, including body weight, weight gain, feed consumption, feed efficiency, serum proteins, liver protein, meat quality (i.e., lipid profile and cholesterol content), antioxidant activity, and lipids’ oxidative stability. For this study, *Sargassum*, *Spirulina*, and *Gracilaria* sp. were purchased. The hypothesized speculation is that algal inclusions would improve the previously mentioned parameters due to their content of antioxidants, vitamin, minerals, and other effective ingredients.

## 2. Materials and Methods

### 2.1. Animal Welfare, Birds, Housing, and Diets

The Committee of the Environment and Life Sciences Research Center at Kuwait Institute for Scientific Research has approved the research proposal of the current study under project No. FA127C (2017). This protocol adheres to the official animal welfare procedures and regulations outlined in reference No. PMO/PV/GM/073/2015.

A total of 1190 one-day-old Cobb 500 broiler chicks were purchased, raised, and fed experimental diets. The chicks were provided with a starter diet from hatching until seven days (1 week) of age, followed by a grower diet from 8 to 21 days (2 to 3 weeks) of age, and a finisher diet from 22 to 35 days (4 to 5 weeks) of age. The feed rations were formulated according to Cobb 500 guidelines using corn and soy [35]. The experimental diet was administered from day one until the chickens were slaughtered at 35 days of age, with water and feed provided ad libitum throughout this study. In the first three days of the brooding period, the one-day-old chicks were provided with 24 h of light in order to give them enough time to find out feed and water. After that, a step down lighting program was followed. Artificial bulbs were used as a source of light. The newborn chicks were kept at a temperature of 33 °C, with a gradual reduction of 3 °C per week until the normal temperature of approximately 24 °C was reached at the beginning of the fourth week and thereafter until the end of the cycle. The humidity was maintained between 50 and 70% throughout the broiler cycle.

Seven dietary treatments were randomly assigned to seven batteries, with 85 birds allocated per battery. Each battery involved 10 levels. Hence, each treatment was replicated ten times (17 birds/replicate). All the seven batteries were housed in a single room. The seven dietary treatments were decided based on the results of the proximate analysis and they were as follows: TRT1, the control group that was fed soybean basal diet without added algae; TRT2 received the same basal diet TRT1 with a 1.0% inclusion of *Sargassum* sp.; TRT3 had a 2.0% inclusion of *Sargassum* sp. along with the basal diet TRT1; TRT4 was given the basal diet TRT1 with a 0.5% inclusion of *Gracilaria* sp.; TRT5 included a 1.0% addition of *Gracilaria* sp. with TRT1; TRT6 received the basal diet TRT1 with a 5.0% inclusion of *Spirulina* sp.; and TRT7 had a 7.5% inclusion of *Spirulina* sp. along with TRT1. The algae were purchased and certified from Farm Ocean Technologies India PVT. Ltd. Nagercoil, Tamil Nadu, India as powdered species. *Sargassum* sp. and *Gracilaria* sp. were produced using raft culture and *Spirulina* sp. was produced using open raceway ponds. Formulation and chemical analyses of the different dietary treatment are available on request from the corresponding author.

### 2.2. Proximate Analyses of the Broiler Feed Rations with the Different Algal Inclusions

Broiler feed rations were prepared with the different algal inclusions and were analyzed for proximate analysis, this includes crude protein, ash, moisture content, and crude fat [6]. Crude protein was determined using the Macro-Kjeldahl method (using Macro-Kjeldahl apparatus, Witeg Labortechnik, Wertheim am Main, Germany), which involves converting nitrogen into ammonium sulfate through acid digestion with boiling sulfuric acid. For ash determination, a known weight of the sample was ignited to a constant temperature, to burn off all the organic materials. The residue left after ignition was the ash. To determine the moisture content, a known weight of the sample was heated to a constant temperature to evaporate all the water. The weight loss of the sample after heating was multiplied by 100 and expressed as the percentage of moisture in the sample. For crude fat determination, an organic solvent was used to extract the crude fat from a known weight of the sample. The solvent dissolved the fat particles present in the sample, and the extracted fat represented the crude fat content. For crude fiber analysis, a sample free of moisture and fat was initially treated with weak acid and base solutions. The remaining residue was collected in a filter crucible, dried, and then ignited. The weight loss after ignition indicated the crude fiber content.

### 2.3. Performance Production Parameters

The parameters assessed in this study included growth rate, feed efficiency, feed in-take, mortality, as well as the temperature and relative humidity of the poultry room. Broiler chicks were regularly weighed at hatch, after one week, and at the end of every two weeks until the end of the cycle. Feed consumption was measured, and feed efficiency was calculated accordingly. Mortality was recorded daily, with the percentage of mortality calculated on a weekly basis.

### 2.4. Serum Collection for Total Serum Proteins S and P and Liver Fatty Acid Binding Protein Analyses

Serum samples of the broiler chickens were collected on weeks three and five of the cycle. All samples were collected in triplicates from the brachial veins of the birds using vacutainer tubes with K2EDTA. Each tube contained 8 to 10 mL of blood, gathered from five randomly selected chickens per treatment. Chicken total serum proteins S and P and liver fatty acid binding protein were measured using commercial enzyme-linked immunosorbent assay (ELISA) kits of LifeSpan BioSciences, Inc., Seattle, WA, USA [36]. Ten birds from each treatment were used in each analysis and samples from each birds were taken in triplicate. Analysis for each sample was performed in triplicate.

### 2.5. Lipid Profile and Cholesterol Content in the Produced Meat Samples

Lipid composition of meat samples in broilers fed the different algal inclusions were investigated using gas chromatography (GC, Hewlett Packard HP 5890 Series, London, UK) [37]. The breast meat samples were analyzed in triplicates. The fatty acid methyl ester (FAME) was identified by matching the retention time of fatty acids beside a known standard (Supelco 37 component FAME mix and PUFA-3 Menhaden Oil standards, Sigma Aldrich, Catalog no. CRM47885). An Agilent 7683 series autosampler was used (SP^®^-2560 Capillary GC Column, L × I.D. 100 m × 0.25 mm, df 0.20 µm, Sigma Aldrich, St. Louis, MO, USA). Chromatograms were plotted and analyzed using the HP ChemStation software package (Hewlett-Packard Co., Reading, UK) [2,38].

Cholesterol concentration in meat samples was measured using commercial ELISA kits (Sigma Aldrich, Gillingham, UK). Breast meat samples were collected at 5- weeks of age from ten birds from each of the seven treatments. Analysis was performed in triplicate. The meat tissue was first rinsed with phosphate-buffered saline (PBS) with a pH of 7.4, to get rid of excess blood and debris, and then it was weighed and homogenized by mincing in PBS. Then, the homogenized solution was centrifuged at 2000−3000 rpm for 20 min. The supernatant was used in the ELISA assay. The Sandwich Kit was pre-coated with chicken antibody. The standard solutions and different dilutions were prepared. A total of 120 µL of the given standard (19.2 mmol/L) was mixed with 120 µL of the standard diluent to generate the desired standard stock solution (9.6 mmol/L). Duplicate standards (0.6, 1.2, 2.4, 4.8 mmol/L) were aliquoted by serial dilution from the same standard stock solution. In the 96-well plate, 50 µL of the respective standards were added into each well. Then, 40 µL of each sample was added to sample wells, followed by the addition of 10 µL of biotin-conjugate anti-chicken antibody into sample well only, not in standard wells. Next, 50 µL of streptavidin-HRP was added to sample and standard wells, carefully avoiding the blank control well. The reagents were then mixed well; the plate was covered with a sealer and then incubated for 60 min at 37 °C. Post incubation, the plate was washed 5 times with wash buffer and 50 µL of stop solution was added to each well; the blue color immediately turned to yellow. Lastly, the optical density (OD value) was determined at 450 nm within 10 min after adding the stop solution, using a microplate reader. The standard curve was obtained by plotting the average OD on the vertical axis (Y) against the concentration on the horizontal axis (X). These calculations can be performed with computer-based curve –fitting software and the best fit line can be determined by regression analysis.

### 2.6. Biochemical Analysis of the Produced Meat Samples

Breast meat samples were harvested from ten random birds from each dietary treatment. Meat samples were analyzed for crude protein, crude fat, fiber, ash, and moisture using MacroKjeldahl and Weende Fiber Tec system methods [39].

### 2.7. Organoleptic Study of the Produced Meat Samples

The meat was cooked with minimal spices to ensure it was edible and acceptable to the panelists. Sensory evaluation parameters included acceptability and palatability tests of the poultry meat, which were assessed through taste paneling and visual observations [40]. A random 20 panelists were provided with a score sheet featuring a 9-point hedonic scale to evaluate each sample from the dietary treatments. Bottled water was provided to cleanse their mouths after tasting each sample. The panelists were given verbal instructions about the score sheet and the test before they began the procedure. Each group of meat samples was labeled with a randomly assigned two-digit code number. The samples were presented in a randomized order, and the panelists were asked to assign scores for color, smell, texture, taste, and general acceptability using a structured nine-point hedonic scale (where 1 indicated the worst quality and 9 indicated the best quality). In this method, the stimuli (i.e., meat samples) were presented individually and were rated on a scale, where the nine categories ranged as follows: 1 = dislike extremely; 2 = dislike very much; 3 = dislike moderately; 4 = dislike slightly; 5 = neither like nor dislike; 6 = like slightly; 7 = like moderately; 8 = like very much; 9 = like extremely. The surveys presented to panelists included brief instructions and a short definition of the study attributes, namely, color, texture, flavor, appearance, smell, taste, and overall acceptability. The testing was conducted in a laboratory at 24 °C under normal lighting conditions to minimize visual differences. The sessions were scheduled from 10:00 a.m. to 1:00 p.m.

### 2.8. Antioxidant Levels in Serum, Liver, and Breast Tissue

Total superoxide dismutase (TSOD) activity in liver was measured using Ransod kit from Randox Laboratories, Crumlin, UK, as described by Habibi et al. [41]. Liver glutathione peroxidase (GPx) was specified based on the protocol of Paglia et al. [42]. The activity of catalase (CAT) enzyme in liver was regulated using the method of Aebi [43]. Malondialdehyde (MDA), superoxide dismutase (SOD), and total antioxidant capacity (TAC) were measured based on the protocols of Habibi et al. [41] and El-Bahr et al. [12].

### 2.9. Oxidation Stability of the Feed Rations with Different Algal Inclusions

Lipid peroxidation of the dietary feed rations, including various concentrations of different algal inclusions, was assessed by measuring the peroxide value (PV) and acid value (AV) over a period of 60 days, starting from Day 10 and continuing every 10 days until Day 60. The PV and AV were measured using the methods outlined by AOCS [44] and Rao et al. [45], respectively.

### 2.10. Statistical Analysis

A total of 1190 birds were randomly allocated into seven dietary treatments. For each treatment, 85 birds were housed in four multi-floor batteries with ten levels each, each level is considered as a replicate, n = 10. Each level had 17 birds. The overall differences between the seven dietary treatments were analyzed using ANOVA, and the general linear model procedure of Minitab v19 was applied. Differences between treatment groups were considered statistically significant at *p* ≤ 0.05. When significant differences were found (*p* ≤ 0.05), pairwise comparisons of treatment means were conducted using the Bonferroni test.

## 3. Results

### 3.1. Proximate Analysis of Starter, Grower, and Finisher Feed Rations with the Different Algal Inclusions

The results of the proximate analyses of the starter, grower, and finisher broiler feed rations with included *Sargassum* sp. at 1.0 and 2.0%, *Gracilaria* sp. at 0.5 and 1%, and *Spirulina* sp. at 5 and 7.5% showed that the different dietary treatments contained acceptable amounts of crude protein, to be used as poultry feed rations. For the starter phase, the crude protein of the 1.0% *Sargassum*-, 2.0% *Sargassum*-, 1.0% *Gracilaria*-, 0.5% *Gracilaria*-, 5.0 *Spirulina*-, and 7.5% *Spirulina*-enriched diets was 19.36, 20.55, 23.80, 23.18, 26.63, and 26.64%, respectively. For the grower phase, the crude protein of the 1.0% *Sargassum*-, 2.0% *Sargassum*-, 1.0% *Gracilaria*-, 0.5% *Gracilaria*-, 5.0 *Spirulina*-, and 7.5% *Spirulina*-enriched diets was 20.99, 21.86, 20.77, 22.27, 24.70, and 24.48%, respectively. For the finisher phase, the crude protein of the 1.0% *Sargassum*-, 2.0% *Sargassum*-, 1.0% *Gracilaria*-, 0.5% *Gracilaria*-, 5.0 *Spirulina*-, and 7.5% *Spirulina*-enriched diets are 19.73, 18.20, 22.27, 19.42, and 22.19%, respectively. The table showing the proximate analysis of starter, grower, and finisher broiler feed rations containing the different algal inclusions is not shown in this paper and was included in the Supplementary Materials.

### 3.2. Effect of Different Algal Inclusions on the Productive Performance of the Broiler Chickens

The impact of incorporating *Sargassum* sp. into the diet on production parameters such as body weight, body weight gain, feed intake, and feed efficiency in broiler chickens was investigated. The results showed no significant effect of the inclusion of varying levels of *Sargassum* sp. on these parameters at different ages, and hence, the table is not included. However, at week 2, there was a borderline effect of using *Sargassum* sp. on body weight gain of broiler chickens (*p* = 0.057). Body weight gain for birds that received 2% of *Sargassum* sp. inclusion had numerically higher body weight gain than those that received 1% of *Sargassum* sp. inclusion, which in turn had a higher body weight gain than the group that received the control diet, but this effect failed to reach significance.

The effect of dietary inclusion of *Gracilaria* sp. and *Spirulina* sp. on body weight, body weight gain, feed consumption, feed efficiency, overall body weight, overall body weight gain, overall feed consumption, and overall feed efficiency of broiler chickens were studied. The results suggested no significant effect of the inclusions of varying levels of the algae.

### 3.3. Effect of Algal Inclusions on Total Serum Protein (TSP) Measurements

The effect of distinctive levels of *Sargassum* sp. at 1.0% and 2.0% on the total serum proteins S and C in three- and five-week-old broiler chickens is studied. The results show that enriching broiler diets with 1% *Sargassum* sp. results in higher concentrations of TSP-S at five weeks of age than the control group and the group enriched with 2% inclusion (*p* = 0.005). The same trend of the effect of 1% *Sargassum* sp. inclusion was noticed in the case of total serum protein-C, but it failed to reach significance (*p* = 0.060).

The effect of different levels of *Gracilaria* sp. at 0.5% and 1.0% on the total serum proteins S and C in three and five-week-old broiler chickens showed that at three weeks of age, diets enriched with 0.5 and 1% *Gracilaria* sp. resulted in lower total serum protein-C than the control (*p* = 0.022). However, there was no significant effect in the case of total serum protein-S. At five weeks of age, the results showed that there was no significant effect of the *Gracilaria* sp. inclusions on the total serum protein. However, numerical analysis showed increased total serum protein-S and total serum protein-C after feeding the broilers with *Gracilaria* sp. inclusions at 0.5 and 1%, but it failed to reach significance.

The effect of different concentrations of *Spirulina* sp. at 5.0% and 7.5% on the total serum proteins S and C at three and five weeks of age of broilers. The results indicate that at three weeks of age, there is no significant effect of the dietary treatments on the total serum proteins. However, at five weeks of age, enriching broiler diets with 5% commercial *Spirulina* sp. resulted in a higher concentration of total serum protein-C than the control group and the group enriched with 7.5% inclusion (*p* = 0.026). No significant effect was observed in the case of total serum protein-S, although numerical analysis showed an enhanced effect after enriching the diet with 5 and 7.5% of *Spirulina* sp. inclusions, when compared to the control group (*p* = 0.151). The table including the results of the effect of algal inclusions on total serum protein (TSP) measurements is included in the Supplementary Materials.

### 3.4. Effect of Algal Inclusions on Liver Fatty Acid Binding Protein (LFABP)

The effect of different dietary treatments on LFABP in three- and five-week-old broilers is shown in Table 1. The results in Table 1 show that supplementing broiler diets with *Sargassum* sp. at 1% and 2% significantly enhances LFABP, when compared to the control in three-week-old broilers. There was no significant effect of the dietary treatments in five-week-old broiler chickens.

The effect of different dietary treatments of *Gracilaria* sp. and *Spirulina* sp. on chicken antibodies in three- and five week-old broilers is shown in Table 1. The results in Table 1 show no significant effect of the dietary treatments on the LFABP of broiler chickens.

### 3.5. Effect of Algal Inclusions on Lipid Profile and Cholesterol Content of the Produced Meat Samples

Table 2 shows the effects of 1.0 and 2.0% of *Sargassum* sp. inclusion on the fatty acid profile of breast tissue in broiler chickens. The results in Table 2 show that the monounsaturated fatty acid, heptadecenoic acid (C17:1), was the highest in the control group rather than the supplemented groups. However, oleic acid (C18:1n9trans) was the highest in the group supplemented with 1.0% *Sargassum* sp., followed by the one supplemented with 2.0%. The control group was the lowest with oleic acid. Results also show that arachidic acid (C20:0), (C18:2n6 trans), γ-Linolenic acid (C18:3n6), dihomo-gamma linolenic acid (C20:3n6), tricosanoic acid (C23:0), and alpha-linolenic acid (C18:3n3) were significantly higher in the control group than the other algae-supplemented groups. Eicosenoic acid (C20:1n9) was higher in the control group, followed by the group supplemented with 2.0% of *Sargassum* sp. Breast samples from the group supplemented with 2.0% of *Sargassum* sp. had the highest level of heneicosylicacid (C21:0), compared to the other groups. Behenic acid (C22:0) was higher in the algae-supplemented groups than the control group. The highest proportion of ∑n-6 was observed in the group of birds that received the control diet. On the other hand, the proportion of ∑n-3 was higher in the algae-supplemented groups than the control group. Most importantly is the ratio of ∑n-6:∑n-3 that reflects the presence of the beneficial fatty acids in the samples. The results show that supplementing broiler feed rations with *Sargassum* sp. significantly lowered ∑n-6:∑n-3 ratio from 16.36 for the control group to 1.53 and 1.79 for the groups supplemented with 1.0 and 2.0% of *Sargassum* sp., respectively (*p* = 0.001).

Table 3 shows the effects of 0.5 and1.0% of *Gracilaria* sp. On the fatty acid profile of breast tissue in broiler chickens. The results in Table 3 show that the proportion of C16:0, C17:1, and C18:3n6 were higher in the control group than the algae-supplemented groups. The proportion of ∑n-3 was significantly higher in the algae-supplemented groups rather than in the control group. Interestingly, this makes the ∑n-6:∑n-3 ratio in the algae-supplemented groups much less than the control group (*p* = 0.037).

Table 4 shows the effect of 5.0 and 7.5% *Spirulina* sp. inclusion on the fatty acid profile of breast tissue in broiler chickens. There was no significant effect of *Spirulina* sp. supplementation on the fatty acids profile of the breast tissue in 5-week-old broiler chickens, except for the n-6:n-3 ratio. Numerically, the total n-3 fatty acids were observed to be the lowest in broilers supplemented with the control diet and the highest was observed with the 7.5% *Spirulina* sp. diet, but this failed to reach significance. As a result of dietary supplementation, the n-6:n-3 ratio decreased 0.60-fold with 5% *Spirulina* sp. and 0.46-fold with 7.5% *Spirulina* sp. in the breast muscle, compared to the control diet (*p* = 0.034).

### 3.6. Effect of Algal Inclusions on Cholesterol Concentration

The effect of different dietary algal treatments on HDL and VLDL in three- and five-week-old broiler chickens was investigated. Results showed that there is no significant effect of *Sargassum* sp. and *Gracilaria* sp. algal inclusions on the cholesterol content in three- and five-wks-old broilers. However, supplementing broiler chickens with *Spirulina* at 5% and 7.5% significantly increased the HDL concentration at 5 weeks of age, compared to the control group. No significant effect was observed in the VLDL concentration (Table 5).

### 3.7. Effect of Sargassum sp., Gracilaria sp., and Spirulina sp. on Biochemical Analysis of the Produced Meat Samples

The proximate analysis of meat samples for birds fed on diets enriched with different algal inclusions was investigated. The results showed that enriching broiler feed rations with *Sargassum* sp. at 1% and 2% reduced the crude fat in the breast tissue samples (*p* = 0.037) from 7.400% for the control group to 5.68% and 6.4% for the group fed 1% and 2% *Sargassum* sp., respectively. No significant difference was observed for the other proximate analyses using *Gracilaria* sp. and *Spirulina* on the proportions of crude protein, ash, and moisture in breast samples.

### 3.8. Organoleptic Study of the Produced Meat Samples

**Figure 1:**
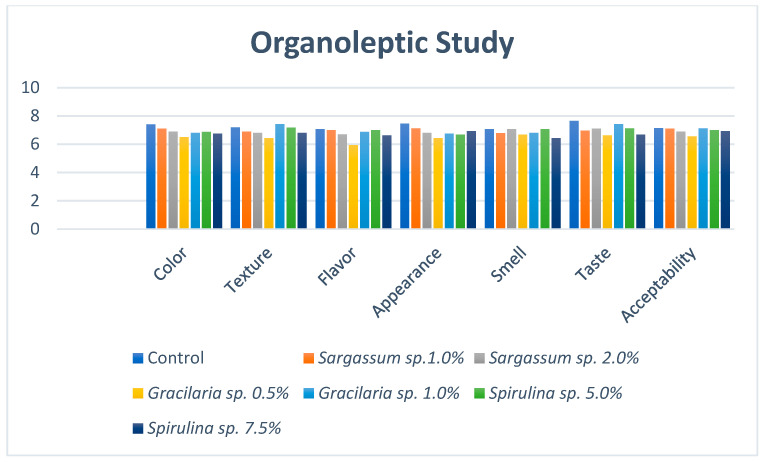
Graphical representation of the organoleptic study of the produced meat samples.

The organoleptic evaluation of meat samples from broilers fed different algal inclusions was studied and the reports from the panelists showed no significant difference in the taste panel parameters such as color, texture, flavor, appearance, smell, taste, and overall acceptance for the meat samples among the groups fed the different dietary treatments (data are shown in the Supplementary Materials) (Figure 1).

### 3.9. Antioxidant Status of Sera, Liver, and Breast Tissue

The antioxidant indices in the serum, liver, and breast tissue of broiler chickens that were fed different concentrations of algae were studied. The results indicate that adding various types of algae at different concentrations to broiler feed significantly enhanced TAC and TSOD levels in serum samples (*p* = 0.034 and 0.045, respectively). The highest TAC content in serum was observed in the group fed 7.5% *Spirulina* sp., where TAC con-centration was four times higher than that in the control group [19]. The highest sera TSOD was observed for the group fed with *Spirulina* sp. at 5% and 7.5%. Broilers enriched with *Spirulina* sp. at 7.5% led to the highest rise in both TAC and TSOD concentrations in all serum samples. With respect to serum MDA, the overall various algal inclusions sig-nificantly reduced MDA levels (*p* = 0.014). This study builds upon one of my previously conducted investigations, Al-Khalaifah [19], (Table 11 in [19]), providing a foundational framework for the current analysis.

Moreover, the inclusion of algae in feed has a significant effect on the activity of liver GPx in the experimental group (*p* = 0.039). All types of algal treatments significantly in-creased the liver GPx activity levels, in comparison to the control group. The highest liver GPx contents were observed in the experimental group treated with *Sargassum* sp. at 2% and in the one enriched with *Gracilaria* sp. at 0.5%. No significant difference was observed in liver CAT levels among broiler chickens fed different dietary algal inclusions (*p* = 0.352). However, it also shows that the liver MDA levels in the control group were significantly higher than those in all other dietary treatments (*p* < 0.001). Compared to the control group, inclusion with *Spirulina* sp. at 5% resulted in the most significant reduction in liver MDA concentration among the treatment groups. Furthermore, Table 11 in [19] shows that enriching broiler feed rations with the different algae at different concentrations significantly increased the breast muscle SOD (*p* = 0.013). In addition, breast tissue MDA was significantly reduced by algal inclusion at different concentrations (*p* = 0.002) in comparison to the control group, with the greatest reduction being observed in the group fed with *Spirulina* sp. at 7.5% of diet.

### 3.10. Oxidation Stability of the Feed Rations with Different Algal Inclusions

Table 6 shows the peroxide value (PV) of lipid extracted from stored feed rations with algal inclusions over time. The results reveal that in the control samples, the PV increases from 13.21 to 25.02 over the first 40 days, and then decreases to 20.35 and 16.56 at days 50 and 60, respectively. A similar trend of initial increase until 40 days, followed by a decrease at day 50 and thereafter, was observed for the other feed ration samples enriched with various algal inclusions.

Table 7 presents the acid value (AV) measurements, showing the effect of algal inclusions on lipid oxidation of stored feed rations over 10-day intervals for 60 days. The results indicate a significant reduction (*p* < 0.001) in AV for all algal treatments on days 10, 20, and 30 compared to the control. During these measurement days, the results indicated that *Spirulina* at 7.5% diet had the most overall significant reduction in the AV levels among all algal treatments. In addition, significant differences in the AV content have been shown between the control and treatment groups after 40 days of storage (*p* = 0.038). Even though an overall significant difference between the treatment groups and control was shown after 50 days of storage (*p* = 0.047), only commercial *Spirulina* sp. at 7.5% group had a significant difference with the control and other algal groups. Additionally, the treatments indicate a non-significant effect after 60 days of storage time (*p* = 0.341).

## 4. Discussion

The current study aims to evaluate the effects of different inclusions of *Sargassum* sp., *Gracilaria* sp., and *Spirulina* sp., on the productive performance, serum proteins, liver proteins, meat quality, and antioxidant activity in broiler chickens. Different proportions of *Sargassum* sp., *Gracilaria* sp., and *Spirulina* sp. were selected based on the algae proximate analyses before formulating the broiler feed rations.

The formulated feed rations including the different proportions of algal inclusions were analyzed for proximate analyses before the feeding trial, to ensure their suitability in fulfilling the nutritional requirements of the broiler chickens at different stages. These analyses included crude protein, crude fat, crude fiber, ash, and moisture. The results of the feed rations proximate analyses revealed that the formulated diets contained the required amounts of protein, fat, fiber, ash, and moisture, to be fed to the broiler chickens. In other words, it was observed that the proximate analysis of all the feed rations containing the different levels and types of marine algae fell within the expected normal ranges [46].

The present results revealed that there was no negative effect of using the different algal inclusions on the production performance parameters of the birds. All the supplemented birds performed normally as the control group. This is in agreement with other studies in the literature that have investigated the effect of algal supplementation in broiler diets. For example, Fan et al. [47] investigated the effect of different dosages of *Sargassum* sp. on the daily feed intake of Leghorn layers (Hy-Line W-36) at 36 weeks of age and concluded that supplementing the laying hens’ diet with a range of 1 to 5% of *Sargassum* sp. did not affect the daily feed intake in the hens. In addition, Lum et al. [48] reported no effect of supplementing broiler chickens with 8% *Spirulina* sp. on body weight, liver weight, abdominal fat, or kidney weight at 16 days of age. On the other hand, it was reported that when three-week-old broiler chickens were supplemented with 20% blue-green algae in their diets, they experienced depressed growth when algal inclusion levels were higher than 10%. At 12% inclusion level, broilers showed a slower growth rate than those fed with 0, 1.5. 3.0, or 6.0% algae in the diet [49].

On the contrary, Khan et al. [50] investigated 120 day-old chicks, assigned to four dietary treatments with 0, 1, 1.5, and 2 g of *Spirulina* sp./kg feed, and reported that dietary supplementation of *Spirulina* sp. improved feed intake (8.95%), weight gain (12.5%), feed conversion ratio, and dressing percentage, compared to the control. Fathi [51] investigated the effect of different levels of *Spirulina* platensis (0.3, 0.5, 0.7, and 0.9 g/kg) on productive performance in broiler chickens. The author concluded that the dietary supplementation with 0.7 and 0.9 g *Spirulina* platensis/kg of feed could improve the growth performance, blood parameters, biochemical changes in serum, and microbial load.

Interestingly, the results of the current study show that at week 2, there was a borderline effect of using *Sargassum* sp. on body weight gain of broiler chickens (*p* = 0.057). Body weight gain for birds that received 2% of *Sargassum* sp. inclusion had numerically higher body weight gain than those that received 1% of *Sargassum* sp. inclusion, which in turn had higher body weight gain than the group that received the control diet, but this effect failed to reach significance.

Blood proteins in birds are crucial indicators for evaluating both health and production performance parameters. They also reflect the general biochemical processes responsible for metabolic alterations in the bird. Besides pathological conditions, several physiological features, such as breeding, molting, and husbandry, are also related to the level of serum proteins in birds. It has been reported that growth processes and physiological changes influence the intensity of metabolites and induce alterations in the pattern of serum proteins [52,53]. In the current study, it is shown that supplementing broiler diet with 1% *Sargassum* sp. induced an increase in the total serum proteins, compared to the other treatments. On the contrary, *Gracilaria* sp. supplementation reduced total serum proteins, compared to the control group. At five weeks of age, supplementing broiler diets with 5% *Spirulina* sp. resulted in a higher concentration of total serum protein-C than the control group and the group supplemented with 7.5% inclusion.

It is reported that the uptake of unsaturated fatty acids into the cells is triggered by LFABP [54]. Reski et al. [55] investigated the effect of brown seaweed on the liver fat of broiler chickens and found that brown seaweed had an effect on liver fat. One reason for this could be that brown seaweeds are rich in alginate, which binds to bile salts in the intestine. Additionally, alginate cannot be digested by poultry because they lack alginate enzymes. The results of the current study revealed that supplementing the diet of 3-week-old broilers with *Sargassum* sp. at 2% elevated the proportions of LFABP in the serum.

Algae exhibit an intriguing polyunsaturated fatty acid (PUFA) composition, especially concerning n-3 and n-6 PUFAs, which are important for overall health. It is well documented that long-chain n-3 fatty acids play a crucial role in maintaining human health [56,57]. Studies in the literature have reported that supplementing poultry rations with algae modulate the fatty acid profile and enhance the proportions of n-3 PUFA in the produced meat and table eggs, reducing the n-6:n-3 ratio [2,13,58]. Rymer et al. [58] confirmed that algae can be used efficiently as fish oil in the enrichment of poultry meat with n-3 PUFA. Similarly, Long et al. [59] investigated the effect of marine microalgae on the fatty acid deposition in 126 as-hatched male Arbor Acres chicks. The dietary treatments included a control diet, a 1% marine algae (MA) diet, and a 2% MA diet. It was observed from the results of the study that there was a decrease in the n-6/n-3 PUFA ratio, and PUFA/SAT fatty acid ratio in the groups supplemented with the marine microalgae, compared to the control group. This is consistent with the results of the present study, which revealed that the highest proportion of ∑n-6 fatty acids was observed in the group of birds fed the control diet, while the proportion of ∑n-3 fatty acids was higher in the algae-supplemented groups compared to the control group. Most important is the ratio of ∑n-6:∑n-3 that reflects the presence of the beneficial fatty acids in the samples. The present results show that supplementing broiler feed rations with marine algae significantly lowered ∑n-6:∑n-3 ratio.

Kumar [27] demonstrated that supplementation with *Sargassum* sp. in broiler diets affected blood cholesterol levels. In birds supplemented with 1% and 2% *Sargassum* sp., there was a reduction in blood plasma cholesterol and globulins, along with improvements in total serum proteins, albumin, calcium, phosphorus, and triglyceride levels. In comparison to controls, *Sargassum* improved dietary palatability while also increasing feed efficiency and improving digestibility and intestinal absorption, resulting in better body weight gain. It is suggested that the active constituents in *Sargassum*, such as saponins, hemicelluloses, mucilage, tannins, and pectin, may influence changes in blood low-density lipoprotein (LDL) cholesterol by blocking bile salts [27]. Yvonne [25] reported that marine algae possess hypocholesterolemic and hypolipidemic properties that induce reduction in the cholesterol content in chickens. In addition, Mirzaie et al. [60] reported that there was a significant reduction in the serum levels of cholesterol, triglycerides, and total lipids when broilers were supplemented with *Spirulina* sp. Brown algae can also reduce LDL cholesterol levels due to their high content of fiber, sterols, and other bioactive compounds with antioxidant properties [61]. Specifically, *Gracilaria* sp. fiber acts as an anticoagulant, anti-hyperlipidemic, antitumor, antiviral, and anti-cholesterol agent, thereby helping to lower LDL concentrations in the blood [62]. Studies have also shown that green, brown, and red seaweed can reduce the concentration of serum total cholesterol, LDL, VLDL, triglycerides and total lipids, and amplified the HDL cholesterol [63]. The results of the current study showed that supplementing broiler chickens with *Spirulina* at 5% and 7.5% significantly increased the HDL concentration, compared to the control group. On the contrary, no effect was observed of the other treatments.

The protein content in marine algae varies depending on the species [64,65]. Brown seaweeds generally have lower protein levels, averaging 5–15% of dry weight, while green and red seaweeds tend to have higher protein content, ranging from 10 to30%. Some red seaweeds, such as *Palmaria palmata* and *Porphyra tenera*, can reach protein levels of up to 35% and 47% of the dry matter, respectively, comparable to high-protein plant-based sources like soybeans (about 35% protein content). Due to these notable protein levels, there has been increasing interest in utilizing seaweeds as a novel and valuable source of not only proteins but also lipids, polysaccharides, minerals, vitamins, enzymes, and omega-3 fatty acids [12,36,66]. Many authors have reported that seaweeds improved the productive performance of chickens, reduced their cholesterol content, and acted as an antioxidant [25,67]. It can be added to poultry feed in proportions of up to 2−10% without having any adverse effect on their performance or taste [68,69]. The current results showed that higher protein and lower fat content were found in the broilers supplemented with almost all the algae species. This was expected, considering the negative correlations between the concentrations of protein and fat. This is possibly attributed to the higher level of locomotory activity that favors myogenesis over lipogenesis, which could be a sign of the positive influence of algal supplementation on the nutritive quality of broiler meat. The highest concentration of protein was observed in broilers supplemented with *Spirulina* sp. at 7.5%, compared to the other treatments. The lowest fat concentration was observed in the treatments with *Gracilaria* (0.5%). The high concentration of fiber was observed in the treatments with *Spirulina* sp. (7.5%), compared to the other groups. In addition, the current results show that supplementing broiler feed rations with *Sargassum* sp. at 1 and 2% reduced the crude fat in the breast tissue samples, compared to the control group. Algal supplementation with *Spirulina* sp. at 5% of diet resulted into the lowest fat content in the breast tissue samples, compared to the other group, but this failed to reach significance.

The present results show that there was no significant difference in the taste panel parameters for the meat samples among groups fed the different dietary treatments. In other words, supplementing broiler chickens with different algal inclusions did not adversely affect the palatability of the meat, as no significant differences were observed in parameters such as appearance, smell, color, flavor, texture, and overall acceptability. These findings are consistent with those of Park et al. [70], who studied the effects of *Spirulina* sp. supplementation on breast meat quality and concluded that there was no effect.

Due to their richness in bioactive compounds, the use of macroalgae in poultry feed rations has been suggested for improving the health and antioxidative status of broilers, as well as the quality of poultry products [61]. The current results are in agreement with many studies in the literature investigating the same effect of algal inclusions on the anti-oxidative status in broiler chickens. For example, El-Bahr et al. [12] investigated the effect of 1 g/kg diet of *Chlorella* vulgaris, *Spirulina* platensis, and *Amphora* coffeaformis on the antioxidant status of broiler chickens. The authors reported that all investigated microalgae decreased MDA and PC levels and increased the SOD activities in breast tissue, in comparison to the control. In addition, Liu et al. [13] fed broiler chickens on diets supplemented with 1000, 2500, 4000, 5500, and 7000 mg/kg diet of algae-derived polysaccharides (ADP) from Enteromorpha sp. The authors showed that dietary 1000–7000 mg/kg ADP inclusion improved the average daily gain and feed conversion ratio. The final body weight was improved by the dietary inclusion of 1000 mg/kg. Their results also showed that dietary 2500 mg/kg ADP inclusion increased CAT and liver T-SOD activities; meanwhile, dietary 1000–5500 mg/kg ADP inclusion reduced serum and liver MDA levels. In addition, dietary 2500 and 4000 mg/kg ADP enhanced the villus height of jejunum and ileum. Dietary inclusion of 4000 mg/kg ADP increased CAT activities in the duodenum and T-SOD activities in the jejunum and ileum, while decreasing MDA contents in the duodenum, jejunum, and ileum. On day 35, dietary inclusion of 1000–7000 mg/kg ADP reduced MDA contents in the duodenum and jejunum. Additionally, Mirzaie et al. [60] investigated the effects of *Spirulina* algae at 5 g/kg, 10 g/kg, and 20 g/kg on the oxidative status and performance of broiler chickens. The authors reported that *Spirulina* supplementation at all levels enhanced the oxidative and immune status of broilers. Interestingly, another study investigated the effect of microencapsulated and non-encapsulated *Spirulina*, each at 0.33, 0.66, or 1% of diet on the productive performance and antioxidation status in broiler chickens [71]. The Authors of the study revealed that supplementing broiler feed rations with encapsulated and non-encapsulated *Spirulina* powder at 1% significantly enhanced serum super oxide dismutase activity, which indicated higher antioxidant activity. In a dose-dependent study, Zeweil et al. [72] demonstrated that a diet of 20 g/kg (2%)-*Spirulina platensis* supplementation significantly increased serum GPx and SOD levels, and reduced serum MDA levels in heat-exposed broilers, in comparison to the control group. The study results further showed the beneficial effect of *Spirulina* in counteracting oxidative stress when included in broilers’ diet due to the presence of C-phycocyanin antioxidant in algae. In the same manner, a study by Rizk [63] revealed that supplementing chicken diets with 0.2% of brown seaweed (Phaeophyceae) significantly increased both serum TAC and GPx contents in treated groups in comparison to control. Similarly, researchers in the study found a significant rise in serum SOD in broilers fed with diets of 0.2% red seaweed (Rhodophyceae) inclusion. Guedes et al. [73] reported that both microalgae and macroalgae are rich sources of powerful antioxidant metabolites including carotenoids, xanthophylls, tocopherols, and polysaccharides. The role of these phenolics in lowering the oxidative stress is scientifically well established [74]. Algae polysaccharides, as in *Sargassum* species, have an inhibitory effect on oxidative stress by increasing the antioxidant capacity through the production of more glutathione, induction of superoxide dismutase and GPx activities, and the reduction in reactive oxygen species level [75]. Flavonoid, phenolic acid, and poly phenol found in *Gracilaria* species are likewise antioxidant agents carrying inhibitory effect on oxidative stress. Interestingly, Tufarelli et al. [76] conducted a feeding trial with laying hens using a completely randomized design with a factorial arrangement of 3 × 3. This design included three different dietary levels of horsetail supplementation (0%, 0.25%, and 0.50%) in combination with three levels of *Spirulina* (0%, 1%, and 2%). The overall findings indicated that the combination of horsetail and *Spirulina* had potential for improving the physical parameters of eggs, while *Spirulina* was more effective in enhancing blood traits and oxidative status.

The oxidative stability of the stored diet over time is reflected by the peroxide value (PV) and acid value (AV) indices. The PV measures hydroperoxides, which are primary oxidation byproducts and indicate the initial stages of lipid oxidation. The initial increase in PV until 40 days may be attributed to the rapid formation of peroxides during this period. After 40 days of storage, secondary oxidation byproducts began to appear, causing the primary byproducts to decrease between 40 and 60 days. Conversely, the AV measures aldehyde and ketone byproducts resulting from the breakdown of peroxides. These oxidation byproducts contribute to the rancidity of lipids in the diet [77]. In general, the current study found higher PV and lower AV in diets supplemented with algae compared to the control diet. Over a 50-day period, the PV initially increased until 30 days of storage, after which it returned to levels comparable to the control diet. This suggests that while algae did not impact the production of primary hydroperoxides, they did prevent their conversion into secondary aldehyde and ketone products. The results regarding lipid stability in stored feed rations align with findings from other studies investigating similar effects [78].

Similarly, the acid value (AV) is an important index that measures the concentration of free fatty acids (FFAs) resulting from the enzymatic degradation of lipid molecules, which indicates the degree of rancidity [79]. The study results show a clear decrease in AV levels within the treatment groups compared to the control, indicating reduced formation of FFAs due to lipid hydrolysis when algae are included as feed material or additives in the broiler diet. This underscores the role of macroalgae in preventing lipid oxidative rancidity, as reported by Taheri [80].

Supplementation with *Spirulina* sp. has been shown to improve protein-C levels due to its immune-modulatory and anti-inflammatory effects. A key component in *Spirulina* is phycocyanin, a pigment-protein complex with strong antioxidant properties. Phycocyanin has the ability to enhance immune function and reduce oxidative stress, which can lead to better regulation of the coagulation cascade such as protein-C [81]. In contrast, *Gracilaria* sp., a red algae rich in polysaccharides and fibers, may reduce total serum proteins by influencing the synthesis and turnover of proteins such as albumin, a major contributor to serum protein levels. Alterations in liver function or protein synthesis could lead to a decrease in serum protein concentrations. Additionally, *Gracilaria* may enhance protein excretion through increased gut motility, further contributing to the reduction in serum protein levels [6,82]. These distinct effects on protein-C and total serum proteins can be attributed to the unique biochemical properties of each organism. *Spirulina* sp. supplementation improves protein-C levels by enhancing immune function and oxidative stress, while *Gracilaria* sp. may reduce total serum proteins due to alterations in protein metabolism, gut function, or liver synthesis by protein turnover and absorption processes.

## 5. Conclusions

This study highlights the significant benefits of incorporating *Sargassum* sp., *Gracilaria* sp., and *Spirulina* sp. into broiler chicken diets, which not only improve overall bird health but also enhance the meat quality by increasing its antioxidant properties and oxidative stability. These algae supplements boost vital proteins in the blood and liver, reflecting improved physiological and immune responses. To achieve optimal results, careful consideration of the algae type and inclusion level is essential, with microalgae recommended at 2% and macroalgae between 1% and 5%. *Spirulina*, for example, enhances immune function and antioxidant status, while *Gracilaria* offers gut health benefits and reduces oxidative stress, although high inclusion levels may interfere with protein absorption. Incorporating algae at appropriate levels supports immune health, growth, and feed conversion efficiency while maintaining nutrient absorption. Future research should explore the long-term effects of algae supplementation on poultry health, production, and meat quality, investigate different forms of algae (e.g., dried vs. powdered), and assess the potential synergistic effects with other feed additives like prebiotics or probiotics. Further studies on the cost-effectiveness, sustainability, and feasibility of algae as a replacement for synthetic additives in commercial poultry farming are necessary to determine its practical applications for improving animal welfare and meeting consumer demand for nutritious and sustainable food.

## Figures and Tables

**Table 1:** Effect of different inclusions on chicken liver fatty acid binding protein (LFABP) in three and five-week-old broiler chickens.

Algae	LFABP Concentration
3-weeks	
Control	0.035 ^a^
Commercial *Sargassum* sp. (1.0%)	0.081 ^b^
Commercial *Sargassum* sp. (2.0%)	0.084 ^b^
SE Mean	0.006
*p*-value	0.024

LFABP = Liver Fatty Acid Binding Protein; differences between the treatment groups with different superscripts are statistically different at *p* ≤ 0.05, for each algae separately, n = 10.

**Table 2:** Effect of varying levels of *Sargassum* sp. inclusion on the fatty acid profile of breast tissue in broiler chickens.

*Sargassum* sp. Inclusion (%)					
Fatty Acid Profile	Control	1.0	2.0	SEM	*p*-Value
C14:0	0.4015	0.0000	0.1908	0.1102	0.174
C14:1	0.0360	0.0000	0.0000	0.0207	0.465
C15:0	0.0570	0.0000	0.0000	0.0329	0.465
C16:0	20.909	21.753	23.206	1.6919	0.664
C16:1n7	3.2970	1.7704	0.6680	0.4268	0.050
C17:0	0.1865	0.0000	1.7679	1.0210	0.493
C17:1	0.1445 ^a^	0.0000 ^b^	0.0000 ^b^	0.0083	0.002
C18:0	7.7785	19.5256	13.365	4.9276	0.368
C18:1n9trans	0.1240 ^c^	21.1206 ^a^	14.623 ^b^	2.7131	0.026
C18:1n9cis	34.0865	1.7255	17.350	9.1107	0.183
C18:2n6trans	0.0920 ^a^	0.0000 ^b^	0.0000 ^b^	0.0028	0.001
C18:2n6cis	24.7390	18.2297	14.324	2.9950	0.187
C20:0	0.1510 ^a^	0.0000 ^b^	0.0000 ^b^	0.0034	0.001
C18:3n6	0.2940 ^a^	0.0000 ^b^	0.0000 ^b^	0.0063	0.001
C20:1n9	0.3855 ^a^	0.0000 ^b^	0.6681 ^c^	0.0396	0.003
C18:3n3	1.5370 ^a^	0.0000 ^b^	0.0000 ^b^	0.1177	0.004
C21:0	0.0000 ^b^	0.0000 ^b^	0.7831 ^a^	0.0182	0.001
C20:2	0.2885	0.0000	0.1194	0.0689	0.128
C22:0	0.0510 ^b^	1.9922 ^a^	1.5641 ^a^	0.1633	0.007
C20:3n6	0.3955 ^a^	0.0000 ^b^	0.0000 ^b^	0.0112	0.001
C22:1n9	0.1625	0.0000	0.0000	0.0314	0.055
C20:3n3	0.0000	10.4511	7.6349	2.2161	0.090
C20:4n6	0.0000	0.0000	0.2058	0.1188	0.465
C23:0	1.2695 ^a^	0.0000 ^b^	0.0000 ^b^	0.0972	0.004
C22:2	0.0000	0.0000	0.1090	0.0629	0.465
C24:1n9	0.0000	1.1617	1.3242	0.3721	0.152
C22:6n3	0.0420	1.6948	1.2306	0.5755	0.259
∑SAT	30.8040	43.2716	40.877	3.2895	0.141
∑MONO	38.2360	25.7782	34.633	6.6537	0.485
∑PUFA	27.0995	30.3756	23.3953	0.1318	0.385
∑n-6	25.5205 ^a^	18.2297 ^b^	14.530 ^b^	2.9228	0.016
∑n-3	1.5790 ^b^	12.1459 ^a^	8.8655 ^a^	2.7947	0.015
∑n-6:∑n-3	16.3621 ^a^	1.5337 ^b^	1.7933 ^b^	0.7736	0.001

SEM = Standard error of mean; ∑SAT = Total percentage of saturated fatty acids (C14:0, C15:0, C16:0, C17:0, C18:0, C20:0, C21:0, C22:0, C23:0); ∑MONO = Total percentage of monounsaturated fatty acids (C14:1, C16:1n7, C17:1, C18:1n9trans, C18:1n9cis, C20:1n9, C22:1n9, C24:1n9); PUFA = Polyunsaturated fatty acid; ∑PUFA = Total percentage of polyunsaturated fatty acids (C20:2, C22:2); ∑n-6 = Total percentage of n-6 polyunsaturated fatty acids (C18:2n6trans, C18:2n6cis, C18:3n6, C20:3n6, C20:4n6); ∑n-3 = Total percentage of n-3 polyunsaturated fatty acids (C18:3n3, C20:3n3, C22:6n3); ∑n-6:∑n-3 = Ratio of ∑n-6 to ∑n-3; Values in the same row with different superscripts are significantly different at *p* < 0.05. Some compounds were not detected because they were under the detection limit.

**Table 3:** Effect of different inclusions of *Gracilaria* sp. on fatty acid profile of breast tissue in broiler chicken.

*Gracilaria* sp. Inclusion (%)					
FattyAcidProfile	Control	0.5	1	SEM	*p*-Value
C14:0	0.4115	0.1966	0.1649	0.1486	0.521
C14:1	0.0546	0.0000	0.0000	0.0315	0.465
C16:0	20.3744 ^a^	19.5963 ^b^	18.4196 ^b^	0.2412	0.024
C16:1n7	2.3852	2.2680	0.9821	1.2305	0.701
C17:0	2.6155	0.0000	1.4069	1.6397	0.588
C17:1	0.1498 ^a^	0.0000 ^b^	0.0000 ^b^	0.0079	0.001
C18:0	1.0640	13.1487	8.3806	5.1438	0.372
C18:1n9trans	3.5594	0.0000	4.4874	3.0396	0.601
C18:1n9cis	19.8666	23.2319	23.8112	12.3893	0.971
C18:2n6trans	19.1879	2.5609	2.0697	11.0261	0.533
C18:2n6cis	13.8543	26.4511	28.9030	8.0695	0.464
C20:0	12.6709	0.0000	0.0000	7.2580	0.460
C18:3n6	0.2240 ^a^	0.0000 ^b^	0.0000 ^b^	0.0047	0.001
C20:1n9	0.9719	1.5828	1.7280	0.6182	0.690
C18:3n3	1.0518	0.0000	0.0000	0.6073	0.465
C21:0	0.2218	0.5514	0.6489	0.1464	0.244
C22:0	0.2422	0.7446	0.7769	0.2307	0.323
C20:3n3	0.8660	8.1259	7.0763	3.0972	0.336
C24:1n9	0.1881	1.0705	1.1445	0.4213	0.337
C22:6n3	0.0000	0.2890	0.0000	0.1669	0.465
∑SAT	37.6004	34.2376	29.7978	9.2503	0.844
∑MONO	27.1757	28.1532	32.1532	12.5013	0.957
∑PUFA	35.1840	37.4269	38.0490	2.9804	0.360
∑n-6	33.2661	29.0120	30.9727	3.3880	0.704
∑n-3	1.9178	8.4150	7.0763	3.8714	0.440
∑n-6:∑n-3	28.5018 ^a^	4.6921 ^b^	6.1619 ^b^	11.4013	0.037

SEM = Standard error of mean; PUFA = Polyunsaturated fatty acid; ∑SAT = Total percentage of saturated fatty acids (C14:0, C16:0, C17:0, C18:0, C20:0, C21:0, C22:0); ∑MONO = Total percentage of monounsaturated fatty acids (C14:1, C16:1n7, C17:1, C18:1n9trans, C18:1n9cis, C20:1n9, C24:1n9); ∑n-6 = Total percentage of n-6 polyunsaturated fatty acids (C18:2n6trans, C18:2n6cis, C18:3n6); ∑n-3 = Total percentage of n-3 polyunsaturated fatty acids (C18:3n3, C20:3n3, C22:6n3); ∑n-6:∑n-3 = Ratio of ∑n-6 to ∑n-3; Values within the same row that do not share a common superscript differ significantly at *p* < 0.05. Some compounds were not detected because they were under the detection limit.

**Table 4:** Effect of different inclusions of *Spirulina* sp. on the fatty acid profile of breast tissue in broiler chicken.

*Spirulina* sp. Inclusion (%)					
Fatty Acid Profile	Control	5.0	7.5	SEM	*p*-Value
C16:0	21.1351	23.0870	23.9684	0.9718	0.255
C16:1n7	3.4775	1.8192	0.9689	1.1951	0.428
C18:0	8.5873	10.1990	11.6750	0.6194	0.086
C18:1n9cis	31.9283	29.8113	27.6422	1.3695	0.234
C18:1n9trans	2.3944	2.6702	2.7832	0.3193	0.706
C18:2n6cis	26.9716	26.2477	26.2321	1.6494	0.938
C20:1n9	1.6911	0.8587	0.0000	0.4964	0.199
C20:3n3	3.5141	5.3069	6.7304	1.1131	0.269
C24:1n9	0.3007	0.0000	0.0000	0.1736	0.465
∑SAT	29.7224	33.2860	35.6434	1.3878	0.122
∑MONO	39.7919	35.1594	31.3942	2.8542	0.261
∑PUFA	30.4857	31.5546	32.9625	2.7625	0.603
∑n-6	26.9716	26.2477	26.2321	1.6494	0.938
∑n-3	3.5141	5.3069	6.7304	1.1131	0.269
∑n-6:∑n-3	8.5513 ^a^	5.2027 ^b^	3.9808 ^b^	1.8802	0.034

SEM = Standard error of mean; PUFA = Polyunsaturated fatty acid; ∑SAT = Sum percentage of saturated fatty acids (C16:0, C18:0); ∑MONO = Sum percentage of monounsaturated fatty acids (C16:1n7, C18:1n9cis, C18:1n9trans, C20:1n9, C24:1n9); ∑n-6 = Sum percentage of n-6 polyunsaturated fatty acids (C18:2n6cis); ∑n-3 = Sum percentage of n-3 polyunsaturated fatty acids (C20:3n3); ∑n-6:∑n-3= ratio of ∑n-6 to ∑n-3; Values in the same row with no common superscript differ significantly at *p* < 0.05; Some compounds were not detected because they were under the detection limit.

**Table 5:** Effect of different *Spirulina* algal inclusions on high density lipoprotein (HDL) and very low density lipoprotein (VLDL) in five-week-old broiler chickens.

Treatments/Age	Cholesterol Concentration	
	HDL	VLDL
At 5 weeks of age		
Control	0.043 ^a^	0.076
*Spirulina* sp. (5%)	0.210 ^b^	0.107
*Spirulina* sp. (7.5%)	0.716 ^b^	0.097
SE Mean	0.376	0.044
*p*-value	0.047	0.880

HDL = high-density lipoprotein VLDL = very-low-density lipoprotein Differences between the treatment groups are statistically different at *p* ≤ 0.05, n = 10.

**Table 6:** Peroxide value (PV) of lipid extracted from stored feed rations with algal inclusions over the time of oxidation.

Peroxide Value (PV) (mEq/kg)						
Time (days)						
Treatment (g/kg)	10	20	30	40	50	60
Control	13.21 ^a^	18.01 ^a^	23.55 ^a^	25.02 ^a^	20.35 ^a^	16.56 ^a^
*Sargassum* sp. (1%)	9.54 ^b^	15.12 ^b^	17.20 ^c^	20.00 ^c^	14.89 ^c^	13.01 ^c^
*Sargassum* sp. (2%)	10.99 ^b^	14.00 ^c^	21.98 ^b^	22.34 ^b^	18.01 ^b^	14.56 ^c^
*Spirulina* sp. (5%)	8.52 ^c^	14.21 ^c^	18.01 ^b^	19.00 ^c^	11.98 ^d^	9.54 ^d^
*Spirulina* sp. (7.5%)	9.87 ^b^	14.89 ^c^	17.50 ^c^	20.56 ^b^	12.99 ^d^	11.21 ^d^
*Gracilaria* sp. (0.5%)	10.32 ^b^	15.35 ^b^	18.94 ^b^	19.21 ^c^	15.21 ^c^	14.54 ^c^
*Gracilaria* sp. (1%)	11.99 ^b^	16.21 ^b^	19.01 ^b^	20.01 ^b^	18.54 ^b^	16.31 ^a^
SEM	1.85	1.15	2.03	1.02	1.45	1.20
*p*-value	<0.001	0.035	0.023	<0.001	0.048	<0.001

Mean values within the same column with different superscripts are significantly different (*p* < 0.005).

**Table 7:** Acid values (AV) of lipid extracted from stored feed rations with algal inclusions over the time of oxidation.

Acid Value (AV) (mg KOH/g)						
Time (days)						
Treatment (g/kg)	10	20	30	40	50	60
Control	12.032 ^a^	15.32 ^a^	17.46 ^a^	20.78 ^a^	29.45 ^a^	30.31
*Sargassum* sp. (1%)	11.01 ^b^	7.99 ^b^	10.23 ^b^	9.54 ^d^	29.01 ^a^	29.89
*Sargassum* sp. (2%)	7.54 ^c^	8.12 ^b^	11.98 ^b^	8.99 ^d^	30.12 ^a^	28.99
*Spirulina* sp. (5%)	6.01 ^c^	7.31 ^b^	9.79 ^c^	10.12 ^b^	27.89 ^a^	29.32
*Spirulina* sp. (7.5%)	5.13 ^d^	7.00 ^c^	8.99 ^c^	11.34 ^c^	25.60 ^b^	30.01
*Gracilaria* sp. (0.5%)	6.12 ^cd^	6.99 ^c^	11.23 ^b^	18.98 ^d^	28.04 ^a^	31.01
*Gracilaria* sp. (1%)	5.93 ^cd^	7.12 ^c^	10.87 ^b^	18.99 ^d^	29.01 ^a^	29.79
SEM	2.13	1.12	1.65	1.09	1.45	1.99
*p*-value	<0.001	<0.001	<0.001	0.038	0.047	0.341

Mean values in the same column with different superscripts are significantly different (*p* < 0.005).

## Data Availability

The original contributions presented in this study are included in the article and Supplementary Materials. Further inquiries can be directed to the corresponding author.

## Supplementary Materials

The following supporting information can be downloaded at: https://www.mdpi.com/article/10.3390/life15040670/s1.
