# Supplementary material for: Investigating the Effect of Algal Inclusions in Broiler Chickens
Source: Life (Basel). 2025 Apr 17;15(4):670. doi: 10.3390/life15040670 (PMC12028919; doi:10.3390/life15040670)
Supplement: Supplementary file 1 [file life-15-00670-s001.zip › life-3557299-supplementary.pdf]

Table S1. Proximate Analysis of starter, grower and finisher broiler feed ration containing the different algal inclusions.

	Algae type/treatment								
	***Sargassum* sp.**								
**Analysis**	**control**			**1 %**			**2 %**		
	**Starter**	**Grower**	**Finisher**	**Starter**	**Grower**	**Finisher**	**Starter**	**Grower**	**Finisher**
**Ash**	6.21 ± 0.38	7.37 ± 0.96	6.07 ± 0.44	5.51 ± 0.85	6.94 ± 0.04	5.95 ± 0.43	6.48 ± 0.37	6.43 ± 0.17	6.40 ± 0.07
**Crude Fat**	3.469 ±1.018	4.33±0.82	4.56±0.81	4.271 ± 0.038	4.07±0.54	4.37±0.46	4.42 ±0.605	3.69±0.146	2.57±1.58
**Crude Fiber**	5.46 ± 0.27	6.19 ± 0.46	5.31 ± 0.02	5.18 ± 0.20	4.35 ± 0.53	5.08 ± 0.64	5.07 ± 0.86	5.37 ± 0.28	4.69 ± 0.51
**Crude Protein**	23.69 ±1.95	24.20±1.92	19.04±0.83	19.36 ±0.61	20.99±1.20	19.73±0.62	20.55 ±1.48	21.86±1.08	18.20±0.44
	***Gracilaria* sp.**								
	**control**			**0.5 %**			**1 %**		
	**Starter**	**Grower**	**Finisher**	**Starter**	**Grower**	**Finisher**	**Starter**	**Grower**	**Finisher**
**Ash**	6.21 ± 0.38	7.37 ± 0.96	6.07 ± 0.44	6.75 ± 0.62	7.32 ± 0.18	5.89 ± 0.40	7.24 ± 0.31	7.80 ± 0.29	2.06 ± 0.48
**Crude Fat**	3.47 ±1.018	4.33±0.82	4.56±0.81	1.37 ±0.950	5.26±0.18	4.14±0.14	1.49 ±0.00	4.17±1.24	4.19±0.29
**Crude Fiber**	5.46 ± 0.27	6.19 ± 0.46	5.31 ± 0.02	4.22 ± 0.54	4.43 ± 0.15	6.08 ± 0.16	5.80 ± 0.79	6.18 ± 0.83	5.44 ± 0.00
**Crude Protein**	23.69 ±1.95	24.20±1.92	19.04±0.83	23.18 ±0.20	22.27±0.74	19.13±4.22	23.80 ±1.69	20.77±1.25	19.42±0.59
	***Spirulina* sp.**								
	**control**			**5 %**			**7.5%**		
	**Starter**	**Grower**	**Finisher**	**Starter**	**Grower**	**Finisher**	**Starter**	**Grower**	**Finisher**
**Ash**	6.21 ± 0.38	7.37 ± 0.96	6.07 ± 0.44	5.89 ± 0.71	6.94 ± 0.57	7.74 ± 0.32	6.28 ± 0.09	6.73 ± 0.45	5.84 ± 0.49
**Crude Fat**	3.47 ±1.018	4.33±0.82	4.56±0.81	4.217 ±0.041	5.36±1.02	3.84±0.35	1.944 ±0.561	5.36±0.59	4.24±0.08
**Crude Fiber**	5.46 ± 0.27	6.19 ± 0.46	5.31 ± 0.02	5.37 ± 0.66	4.32 ± 0.36	5.03 ± 0.09	5.01 ± 0.03	4.69 ± 0.04	4.71 ± 0.67
**Crude Protein**	23.69 ±1.95	24.20±1.92	19.04±0.83	26.63 ±2.16	24.70±1.20	22.19±0.36	26.646 ±0.86	24.05±0.25	24.48±1.35

Table S2. Organoleptic evaluation of meat samples from broilers fed different levels of algal inclusions.

	Treatment						
**Parameter**	**Color**	**Texture**	**Flavor**	**Appearance**	**Smell**	**Taste**	**Overall acceptability**
**Control**	7.40	7.20	7.06	7.46	7.06	7.66	7.13
***Sargassum* sp.1.0%**	7.10	6.90	7.00	7.12	6.79	6.97	7.10
***Sargassum* sp. 2.0%**	6.90	6.80	6.70	6.81	7.06	7.11	6.89
***Gracilaria sp.* 0.5%**	6.50	6.43	5.93	6.43	6.68	6.62	6.56
***Gracilaria* sp. 1.0%**	6.81	7.43	6.87	6.75	6.81	7.43	7.12
***Spirulina* sp. 5.0%**	6.87	7.18	7.00	6.68	7.06	7.12	7.00
***Spirulina* sp. 7.5%**	6.75	6.81	6.62	6.93	6.43	6.68	6.93
**SE Mean**	0.44	0.42	0.44	0.43	0.38	0.35	0.45
**P-Value**	0.703	0.481	0.361	0.547	0.745	0.167	0.901
